# Attitude and subjective wellbeing of non-compliant mothers to childhood oral polio vaccine supplemental immunization in Northern Nigeria

**DOI:** 10.1186/s12889-018-5126-1

**Published:** 2018-02-08

**Authors:** Gregory C. Umeh, Terna Ignatius Nomhwange, Anthony F. Shamang, Furera Zakari, Audu I. Musa, Paul M. Dogo, Victor Gugong, Neyu Iliyasu

**Affiliations:** 1 World Health Organization, Field Office Kaduna, Kaduna, Nigeria; 2 Kaduna State Ministry of Health, Kaduna, Nigeria; 3 Emergency Operations Centre (sEOC), Kaduna, Nigeria; 4 Kaduna State Primary Health Care Agency, Kaduna, Nigeria

**Keywords:** Attitude, Subjective well-being, OPV supplemental immunization

## Abstract

**Background:**

Attitude and subjective well-being are important factors in mothers accepting or rejecting Oral Polio Vaccine (OPV) supplemental immunization. The purpose of the study was to determine the role of mothers’ attitude and subjective wellbeing on non-compliance to OPV supplemental immunization in Northern Nigeria.

**Methods:**

The study utilized a cross-sectional design to assess attitude and subjective well-being of mothers using previously validated VACSATC (Vaccine Safety, Attitudes, Training and Communication-10 items) & SUBI (Subjective Well-being Inventory-40 items) measures. A total of 396 participants (equal number of non-compliant and compliant mothers) from 94 non-compliant settlements were interviewed, after informed consent. T-test was run to assess difference in mean scores between the non-compliant and compliant mothers on VACSATC and SUBI measures.

**Results:**

The research showed a significant difference in mean scores between the non-compliant and compliant groups on VACSATC measure of mothers’ attitude (M = 18.9 non-compliant, compared to 26.5 compliant; *p* < 0.05). On subjective well-being, the study showed there was no significant difference in the mean scores of the SUBI measure (M = 77.4 non-compliant, compared to 78.0 compliant; *p* > 0.05).

**Conclusion:**

The research has shown that negative attitude is more commonly present in non-compliant mothers and may be a factor in vaccine refusal in Northern Nigeria.

**Electronic supplementary material:**

The online version of this article (10.1186/s12889-018-5126-1) contains supplementary material, which is available to authorized users.

## Background

Polio is one of the preventable causes of acute flaccid paralysis in childhood, with under 5 years of age children at the greatest risk of contracting it [1–5]. The global effort to eradicate poliomyelitis has gained momentum over the years but three countries, Afghanistan, Pakistan and Nigeria remain endemic for Wild Poliovirus (WPV) [6–9]. The World Health Assembly in 1988 declared war against polio with over 99% global reduction in confirmed cases of WPV in the last 25 years [7, 8]. Poliovirus is transmitted through feco-oral route from infected to non-infected child. It is a vaccine preventable disease, but barriers to effective immunization in many low income countries, for example Nigeria have slowed the march to a polio free world [10–13].

The way a mother thinks has influence on her decision to accept immunization and other health interventions [14–18]. Negative attitude can trigger in mothers non-healthy or maladaptive practices. This may endanger the lives of their children, e.g. non-compliance to supplemental Oral Polio Vaccine (OPV) immunization, neglect of personal hygiene, poor nutritional practices and others [19–21].

Likewise, the way a mother feels with regards to her day-to-day concerns, like her health, welfare or family can influence health promotion and disease prevention behaviors [22]. Those who feel good about themselves are more likely to go an extra mile to protect their children from vaccine preventable diseases. Such mothers will take their children to the health center for Routine Immunization (RI) and will readily avail their children of the benefits of other childhood survival programs, like, supplemental OPV immunization, vitamin A supplementation, de-worming campaign and others [23, 24].

In Nigeria, a child completes Routine Immunization (RI), requiring five contacts with healthcare workers at first year of life, as clearly scheduled in the Expanded Program on Immunization [25]. However RI in Nigeria is fraught with many challenges, including negative attitude to immunization and neglect of health promotion and disease prevention activities, with OPV 3 coverage in Northern Nigeria less than 80% [25, 26]. The failure of RI has led to OPV supplemental campaign, and this strategy has met with stiff resistance and high numbers of non-compliance from parents, clerics and some opinion leaders in many communities in Northern Nigeria [27–30]. The boycott of polio vaccination in mostly northern communities in 2003, due mainly to unsubstantiated safety concerns and politics was a great setback in interrupting WPV circulation in Nigeria [27, 28, 30].

Previous researches in public health and social sciences have studied attitude of parents and healthcare workers to immunization in both developed and low income countries [14–21], but no cross-sectional comparative studies have fully investigated the association between attitude and mothers’ subjective wellbeing with non-compliance to OPV supplemental immunization in Northern Nigeria.

This study compared the attitude and subjective well-being of non-compliant mothers to OPV supplemental immunization and comparable number of compliant mothers living in the same community. The purpose of the study was to determine the association between mothers’ attitude and subjective wellbeing with non-compliance to OPV supplemental immunization in Northern Nigeria.

## Methods

### Participants

Participants for the study were randomly sampled from a line-list of non-compliance households over 3 campaigns of OPV supplemental immunization campaign, and a compliant group of comparable size, socioeconomic status, ethnicity, language, age and setting was also randomly sampled from the same settlement. A non-compliance household being one where a caregiver had refused OPV during at least one supplemental immunization campaign and remained unresolved at the time of this research was eligible. Sampling was conducted in two stages using a random table, firstly a non-compliance settlement was randomly sampled from a line-list of non-compliance settlements, and secondly a household was randomly sampled from the already sampled non-compliance settlement. In situations where a household has more than one mother, one mother was randomly sampled for the study. A settlement with 10 or more non-compliant households is regarded as a non-compliant settlement. Settlements are geo-political entities and the numbers of households vary from settlement to settlement. The study was conducted in Rigasa, an urban community with a mixture of middle class, poor and slum settlements. Rigasa has electricity, pipe borne water and telephone facilities. It is a predominantly Hausa/Fulani community, though other tribes like, Yoruba, Nupe, Ibira are also residents. The inhabitants are mainly traders, artisans, and farmers. Rigasa has a projected population of 184, 628, with women of child bearing age and under five population of 40,618 (22%) and 36,925 (20%) respectively [31] and 2309 settlements, of which 462 (20%) are non-compliant [8]. Rigasa is the highest risk ward (lowest administrative unit of government) in Igabi Local Government Area (LGA) of Kaduna State, Nigeria, using a point ranking assessment system taking into account: key performance, immunity, and epidemiological indicators. The High Risk Analysis (HRA) is the standardized tool for risk assessment of wards/LGAs based on a combined approach using both Global and CDC/National program methodology [32]. The prevalence of non-compliance households in Rigasa from monitoring data over three rounds of OPV supplemental immunization campaigns in 2013 was 15% [8]. The sample size (198 non-compliant and 198 compliant mothers) was estimated using Cochran’s formula, 1963 [33].

The size of participants for the study was estimated as follows:

$$ {\mathrm{n}}_0=\frac{{\mathrm{Z}}^2\mathrm{p}\ \mathrm{q}}{{\mathrm{e}}^2} $$

Cochran’s formula, 1963 [33].

Where: n_0_ = the size of participants.

Z^2^ = abscissa of the normal curve (Z α_/2_ = 1.96), 5% significant level, power 80%.

*P* = prevalence of non-compliance households.

q = 1-p.

e = precision, 5% (95% Confidence Interval)

$$ {\mathrm{n}}_0=\frac{(1.96)^20.15\times 0.85}{(0.05)^2} $$

= 198 × 2 (equal size of study and control groups).

=396 participants.

### Study tool

A cross-sectional observational study and research variables were measured using previously validated VACSATC (Vaccine Safety, Attitudes, Training and Communication) & SUBI (Subjective Wellbeing Inventory) measures. These measures have not been previously used in Nigeria. The measures were back-translated from English to Hausa language, before administration to participants.

Attitude was measured with VACSATC based on 10 core themes: source of information; experience; refusal; doubts; safety; future; trust; satisfaction; health importance and understanding of vaccination [34] (see Additional file 1). The measure has been used in many European countries: Poland, Norway, Sweden, United Kingdom, and Spain-for attitudinal study in immunization, and has a stable content validity and test re-test over time [34]. The items were scored using a 5-Likert scale, and yes & no. The response to each item was assigned a numerical value [34].

Subjective well-being was measured with SUBI, a 40-item measure that assesses 11 factorial dimensions: general well-being-positive affect; expectation-achievement congruence; confidence in coping; transcendence; family group support; social support; primary group concern; inadequate mental mastery; perceived ill-health; deficiency in social contacts and general well-being-negative affect [22] (see Additional file 1). The measure has an extraordinary degree of stability in content of factors and also test retest after 18 months. The items were scored using a 3-Likert scale, the minimum and maximum scores obtained by a subject were 40 and 120 respectively. The response to each item was assigned a numerical value [22].

### Procedure

Participants were interviewed at their homes. They were informed on the objectives, purpose, significance and duration of the research, and their rights and level of involvements. Participants who provided written informed consent completed the back-translated questionnaires, administered by the researcher and his assistants.

### Statistical analysis

Data generated from the study was collected manually and entered electronically, and analyzed with Statistical Package for Social Science version 16 (SPSS). In order to satisfy the 2 objectives of this study paired samples T-test analyses were run. The overall VACSATC scores as well as the scores of the individual components of the VACSATC measure were compared between the non-compliant and compliant groups. Scores of the non-compliant and compliant groups on SUBI measure was also compared. In addition, to test the relationship of the two variables Pearson’s coefficient of correlation was run on the overall scores of VACSATC and SUBI scores. The statistical significance was *p* < 0.05.

### Ethical approval

Ethical approval was obtained from Ethical Review Committee, Kaduna State Ministry of Health, by the researcher to conduct the research.

## Results

### Demographic characteristics of the participants

Participants for the study were non-compliant mothers to at least one round of OPV supplemental immunization, and a compliant group of comparable size, socioeconomic status, ethnicity, language, age and location. An equal number of 198 non-compliant and compliant mothers took part in the study, from 94 out of 462 non-compliant settlements. About 3% of the respondents could not continue with the study because of incomplete data. The ages of the non-compliant group ranged from 19 to 61 years (M = 32.00, SD = 7.94), and the compliant group, 18 to 63 years (M = 32.00, SD = 8.10) (see Table 1). The highest educational attainment among the non-compliant group was tertiary education (*n* = 16, 8.1%), and the compliant group (*n* = 25, 12.6%) (See Table 1). The most dominant tribe in both the non-compliant and compliant groups was Hausa/ Fulani, (*n* = 157, 79.3%) and (*n* = 164, 82.8%) respectively. The majority of the participants were petty traders and full-time housewives, in the non-compliant (28.8%, 47.0%) and compliant group (28.8%, 51.5%) respectively (see Table 1). The study revealed that 97% of the participants earned less than 1 USD per day (see Table 1). Number of children per participant ranged from 1 to 9 (M = 3.84, SD = 1.97), 0 to 10 (M = 3.84, SD = 1.90), in the non-compliant and compliant group respectively.

**Table 1:** Demographic characteristics of the respondents

Characteristics	Non-compliant N (%)	Compliant N (%)	^a^*P*-value
Age (Years)			
< 19	1 (0.5%)	3 (1.5%)	*P* > 0.05
20–29	68 (34.3%)	65 (32.8%)	
30–39	82 (41.5%)	89 (45.0%)	
40–49	42 (21.2%)	30 (15.1%)	
50–59	4 (2.0%)	9 (4.6%)	
> 60	1 (0.5%)	2 (1.0%)	
Level of Education ^b^			
Quaranic	50 (25.3%)	40 (20.2%)	*P* > 0.05
Primary	68 (34.3%)	52 (26.3%)	
Secondary	64 (32.3%)	81 (40.9%)	
Tertiary	16 (8.1%)	25 (12.6%)	
Occupation			
Petty Trader	76 (38.4%)	57 (28.8%)	*P* > 0.05
Business Woman	25 (12.6%)	27 (13.6%)	
Company Worker	1 (0.5%)	3 (1.5%)	
Civil Servant	3 (1.5%)	9 (4.5%)	
House-wife	93 (47.0%)	102 (51.5%)	
Income (USD/Day)			
< 0.15	104 (52.5%)	93 (47.0%)	*P* > 0.05
0.16–0.29	50 (25.3%)	46 (23.2%)	
0.30–0.45	24 (12.1%)	35 (17.7%)	
0.46–0.65	9 (4.5%)	15 (7.6%)	
0.66–0.79	6 (3.0%)	5 (2.5%)	
> 0.80	5 (2.5%)	4 (2.0%)	

^a^ Statistical significance at *p* < 0.05 (2-tailed)

^b^ Quaranic = 2 years schooling; Primary = 6 years; Secondary = 9-12 years; Tertiary = 16 years and above

### Attitude of mothers to childhood OPV supplemental immunization

The research showed a significant difference in mean scores between the non-compliant and compliant group on overall scores on VACSATC 10 items measure of mothers’ attitude to childhood OPV immunization (M = 18.9 non-compliant, compared to 26.5 compliant group; *p* < 0.05).

There was a significant statistical difference in mean scores of eight out of the 10 items of VACSATC measure (see Table 2), except for sources of information and trusted source of information.

**Table 2:** Comparison of mean scores on attitude and subjective well-being

	Non-Compliant	Compliant	^a^*P*-value
VACSATC			
Overall Scores	18.9	26.5	0.001
Source of Information	1.36	1.34	0.757
Trusted Source of Information	1.24	1.30	0.218
Overall Satisfaction with Immunization	2.06	3.97	0.001
Satisfaction with the Way Given	2.29	4.10	0.001
Refusal to Vaccination	1.12	1.78	0.001
Doubt about Immunization	1.11	1.83	0.001
Worries about Vaccination Safety	1.38	1.73	0.001
Vaccination of Your Child in Future	2.99	3.70	0.001
Importance of Vaccination	2.70	4.35	0.001
Seriousness of VPD	2.64	2.40	0.045
SUBI	77.4	78.0	0.558

^a^ Statistical significance at *p* < 0.05 (2-tailed)

Respondents main sources of information about vaccination includes: health worker, radio, television, mosque announcement, traditional leaders, town announcers, Newspapers, posters/leaflets, banners, relatives/neighbors, Voluntary Community Mobilizers (VCM), Polio Survivors Group (PSG). The commonest source of information about vaccination was radio, non-compliant (38.8%) & compliant (39.0%).compliant group (M = 1.36 non-compliant, and 1.34 compliant group; *p* > 0.05).

The most trusted source of information was the health worker in (36.0%) and (38.2%) of non-compliant and compliant groups respectively (M = 1.24 non-compliant, and 1.30 compliant group; *p* > 0.05) (see Table 3).

**Table 3:** Main sources of information about vaccination

Source of information	Non-Compliant N (%)	Compliant N (%)	^a^*p* > 0.05
Health Worker	(77) 38.9%	(74) 37.4%	
Radio	(150) 75.8%	(149)75.3%	
Television	(59) 29.8%	(67)33.8%	
Mosque Announcement	(67) 33.8%	(55) 27.8%	
Poster/Leaflet	(0) 0%	(1)0.5%	
Banner	(0)0%	(2)1.0%	
Relative/Neighbor	(7) 3.5%	(15)7.6%	
VCM ^b^	(8) 4.0%	(12)6.1%	
PSG ^c^	(0) 0%	(1)0.5%	
Town Announcer	(0) 0%	(0) 0%	
Traditional Leader	(11) 5.6%	(2)1.0%	
Newspaper	(6) 3.0%	(6) 3.0%	

^a^ Statistical significance *p* < 0.05 (2-tailed)

^b^ Voluntary Community Mobilizer

^c^ Polio Survivor Group

### Subjective well-being of mothers

On subjective well-being, the study showed there was no significant difference in the mean scores on the 40 items SUBI measure between the non-compliant and compliant groups (M = 77.4 non-compliant, compared to 78.0 compliant; *p* > 0.05).

### Correlation between subjective well-being and mothers’ attitude to OPV supplemental immunization

Pearson’s correlation coefficient showed an insignificant negative relationship between subjective well-being and mothers’ attitude to OPV supplemental immunization (*r* = − 0.006; *p* > 0.05 non-compliant, compared to *r* = − 0.113; *p* > 0.05 compliant).

## Discussion

We found that negative attitude to childhood OPV supplemental immunization was more commonly present among non-compliant mothers to OPV supplemental immunization. Mothers’ negative attitude to OPV supplemental immunization is a likely determinant of non-compliance to childhood OPV immunization in Northern Nigeria. There was no evidence to support that lower subjective well-being was more commonly present among non-compliant mothers to childhood OPV supplemental immunization.

Non-compliant mothers were least satisfied with immunization services and more likely to refuse vaccines offered them due to doubts and worries about vaccine safety. The acceptance of immunization as a useful practice in the prevention of diseases and overall protection of the community was more in the compliant mothers. The non-compliant mothers though not unaware of the seriousness of vaccine preventable diseases were least likely to accept vaccine offered to their children in future (see Table 2). Both the compliant and non-compliant mothers have trust in the health workers and radio, in the delivery of immunization services and information (see Table 3). Anecdotal evidence showed that most households have radio in the homes, and most health related information is disseminated to the people using the local radio stations.

These results are consistent with a similar study on attitude by Stefanoff et al. [34]. Both studies found that health workers were the most trusted source of information and attitude of the respondents were generally positive with regards to childhood immunization. However, differences exist in scope, method of data collection, and the age limits of children whose mothers were interviewed. Stefanoff et al. conducted their study in five countries in Europe; they interviewed either parent of children less than 3 years, and obtained data through oral interviews, telephone interviews and e-mails.

The non-compliant households and the comparable compliant households were sampled from the same settlement. This sampling approach may be responsible for no significant difference between the two groups on subjective well-being scores.

There was no significant relationship between subjective well-being and mothers’ attitude to OPV supplemental immunization in both the non-compliant and compliant groups. This may be due to no difference in mean scores between the two groups on subjective well-being.

This study has shown that attitude of mothers to OPV supplemental immunization is associated with compliance to OPV vaccination.in Northern Nigeria. Negative attitude to OPV supplemental immunization has likely influence on mothers’ immunization seeking behaviors and may contribute to non-compliance to OPV supplemental immunization in Northern Nigeria [20, 21].

### Limitations

The cross-sectional design is a limitation and causality cannot be inferred. The study considered only two variables (attitude and subjective wellbeing) out of many that contribute to mothers’ non-compliance to OPV supplemental immunization. The extent to which the research findings can be replicated in other non-compliant households living in either non-compliant or compliant settlements is limited by its cross-sectional design. The study was conducted in one ward (sub-district), and the findings may not be applicable to other areas in Nigeria, though Rigasa is a typical non-compliant ward in Northern Nigeria. The study looked at the attitude of mothers although it is often the fathers who are opposed to vaccines and do not allow their children to be vaccinated. However, mothers are the ones who interphase with vaccination teams. Though some of the respondents were above 50 years, and may not have < 5 year old children, this does not significantly affect the results of the study as they constitute 6% of the total respondents. Another limitation is the validity of VACSATC, which may be lower in Nigeria, given that it was developed and used in Europe; however the results were statistically significant. The researcher and his assistants offered no counseling or talking therapy to non-compliant mothers, based on the likely reason(s) for their negative attitude.

Non-compliance to childhood OPV supplemental immunization is more common among mothers with negative attitude to OPV supplemental immunization in Northern Nigeria. Although this is limited in scope, future researches should explore factors which are associated with poor attitude towards OPV supplemental immunization.

The government in collaboration with partners and experts should support intervention studies to fully understand factors that may be responsible for non-compliance to childhood OPV supplemental immunization in Northern Nigeria and beyond. These interventions may include one-on-one talking therapy/counseling for a mother or group of mothers in a settlement/community on the benefits, side effects, costs, accessibility and other concerns about childhood OPV supplemental immunization. The counseling/communication should combine behavioral approaches/modified Cognitive Behavioral Therapy (CBT) directed systematically at the core negative attitude through gradual and sequential unraveling of the negative thoughts, biases and dysfunctional assumptions about childhood OPV supplemental immunization rendered by a trained nurse or social worker [35].

## Conclusion

Negative attitude to immunization is more prevalent among non-compliant households/mothers and may be a significant factor leading to vaccine refusal. Counseling or talking therapy for mothers non-compliant to childhood OPV supplemental immunization and future researches to explore other variables in evolution of non-compliance to OPV supplemental immunization will be needed to ensure the march to a polio-free Nigeria is achieved.

## Additional file

Additional file 1: Research Questionnaires. (DOCX 14 kb)

## Abbreviations

CBT: Cognitive behaviour therapy
CDC: Centre for disease and control
HRA: High risk analysis
LGA: Local Government Area
OPV: Oral polio-vaccine
PSG: Polio Survivor Group
RI: Routine immunization
SPSS: Statistical package for social sciences
SUBI: Subjective wellbeing inventory
VACSATC: Vaccine safety, attitudes, training and communication
VCM: Voluntary community mobilizers
WPV: Wild polio virus
