# Supplementary material for: Attitude and subjective wellbeing of non-compliant mothers to childhood oral polio vaccine supplemental immunization in Northern Nigeria
Source: BMC Public Health. 2018 Feb 8;18:231. doi: 10.1186/s12889-018-5126-1 (PMC5806367; doi:10.1186/s12889-018-5126-1)
Supplement: Supplementary file 1 — Research Questionnaires. (DOCX 14 kb) [file 12889_2018_5126_MOESM1_ESM.docx]

**Additional File 1**

Research Questionnaires

| VACSTAC   1. Source of Information: What are your main sources of information about vaccination? 2. Experience: Overall how satisfied were you with your last vaccination? 3. Refusal: Have you ever chosen not to give your child a vaccine that you have been offered? 4. Doubts: Have you ever had doubts about having your child vaccinated? 5. Safety: Have you ever had worries about the safety of a vaccination? 6. Future: Would you have your child vaccinated with vaccines offered to you in future? 7. Trust: Who do you trust the most to give health advice and information about immunization? 8. Satisfaction: Are you satisfied with the way in which vaccination is provided? 9. Health Importance: How important are vaccination in protecting the whole community against diseases? 10. Understanding: How Serious are vaccine preventable diseases to your child?   SUBI   1. Do you feel your life is interesting? 2. Do you think you have achieved the standard of living and the social status you had expected? 3. How do you feel about the extent to which you have achieved success and are getting ahead? 4. Do you normally accomplish what you wanted to? 5. Compare with the past, do you feel your present life is: 6. On the whole, how happy are you with the things you have been doing in recent years? 7. Do you feel you can manage situations even when they do not turn out as expected? 8. Do you feel confident that in the case of a crisis (anything which substantially upsets your life situation) you will be able to cope with it/face it boldly? 9. The way things are going now; do you feel confident in coping with the future? 10. Do you sometimes feel that you and the things around you belong very much together and are integral part of a common force? 11. Do you sometimes experience moments of intense happiness? 12. Do you sometimes experience a joyful feeling of being part of mankind as of one large family? 13. Do you feel confident that relatives and/or friends will help you out if there is an emergency, e.g. if you lose what you have by fire or theft? 14. How do you feel about the relationship you and your children have? 15. Do you feel confident that relatives and/or friends will look after you if you are severely ill or meet with an accident? 16. Do you get easily upset if things don’t turn out as expected? 17. Do you sometimes feel sad without reason? 18. Do you feel too easily irritated, too sensitive? 19. Do you feel disturbed by feelings of anxiety and tensions? 20. Do you consider it a problem for you that you sometimes lose your temper over minor things? 21. Do you consider your family a source of help to you in finding solutions to most of the problems you have? 22. Do you think that most of the members of your family feel closely attached to one another? 23. Do you think that you would be looked after well by your family in case you were seriously ill? 24. Do you feel your life is boring/uninteresting? 25. Do you worry about your future? 26. Do you feel your life is useless? 27. Do you sometimes worry about the relationship you and your wife/husband have? 28. Do you feel your friends/relatives would help you out if you were in need? 29. Do you sometimes worry about the relationship you and your children have? 30. Do you feel that minor things upset you more than necessary? 31. Do you get easily upset if you are criticized? 32. Would you wish to have more friends than you actually have? 33. Do you sometimes feel that you miss a real close friend? 34. Do you sometimes worry about your health? 35. Do you suffer from pains in various parts of your body? 36. Are you disturbed by palpitations/a thumping heart? 37. Are you disturbed by a feeling of giddiness? 38. Do you feel you get tired easily? 39. Are you troubled by disturbed sleep? 40. Do you sometimes worry that you do not have close personal relationship with other people? |
| --- |
